# An Atypical 15q11.2 Microdeletion Not Involving *SNORD116* Resulting in Prader–Willi Syndrome

**DOI:** 10.1155/2023/4225092

**Published:** 2023-09-13

**Authors:** Molly M. Crenshaw, Sharon L. Graw, Dobromir Slavov, Theresa A. Boyle, Daniel G. Piqué, Matthew Taylor, Peter Baker

**Affiliations:** ^1^University of Colorado School of Medicine (CU-SOM), Department of Pediatrics, Section of Genetics and Metabolism, Aurora, Colorado, USA; ^2^Colorado Genetics Laboratory, Department of Pathology, University of Colorado Anschutz Medical Campus, Aurora, Colorado, USA; ^3^Division of Cardiology, Cardiovascular Institute, University of Colorado Anschutz Medical Campus, Aurora, Colorado, USA; ^4^Department of Pathology, Stanford University, Palo Alto, California, USA; ^5^Adult Medical Genetics Program, Department of Medicine, University of Colorado Anschutz Medical Campus, Aurora, Colorado, USA

## Abstract

Loss of expression of paternally imprinted genes in the 15q11.2-q13 chromosomal region leads to the neurodevelopmental disorder Prader–Willi Syndrome (PWS). The PWS critical region contains four paternally expressed protein-coding genes along with small nucleolar RNA (snoRNA) genes under the control of the *SNURF-SNRPN* promoter, including the *SNORD116* snoRNA gene cluster that is implicated in the PWS disease etiology. A 5-7 Mb deletion, maternal uniparental disomy, or an imprinting defect of chromosome 15q affect multiple genes in the PWS critical region, causing PWS. However, the individual contributions of these genes to the PWS phenotype remain elusive. Reports of smaller, atypical deletions may refine the boundaries of the PWS critical region or suggest additional disease-causing mechanisms. We describe an adult female with a classic PWS phenotype due to a 78 kb microdeletion that includes only exons 2 and 3 of *SNURF-SNRPN* with apparently preserved expression of *SNORD116*.

## 1. Introduction

Prader–Willi Syndrome (PWS) is a neurodevelopmental disorder caused by the loss of expression of paternally expressed genes in the 15q11.2-q13 chromosomal region. This loss is most commonly due to a paternally inherited deletion (70–75% of cases) or maternal uniparental disomy (25–30% of cases), and less commonly from imprinting defects, balanced translocations, or rare microdeletions (1%) [1–3]. PWS is relatively rare and is estimated to occur in 1 : 15,000–1 : 25,000 live births [3]. Manifestations of classic PWS include failure-to-thrive and hypotonia, ultimately leading to developmental delay, hyperphagia, and behavioral abnormalities [2, 4, 5].

While the importance of paternally expressed genes in the development of PWS pathology has been appreciated for many years, the identification of specific critical genes associated with the phenotype has proved challenging. Small nucleolar RNA (snoRNA) genes that are under the control of the *SNURF-SNRPN* promoter, namely, the *SNORD116* snoRNA gene cluster, have been implicated directly in disease etiology [3, 6]. This *SNRPN* gene is responsible for encoding the bicistronic *SNURF-SNRPN* transcript, hence why the genes are typically annotated together [2]. Multiple reports of microdeletions including the *SNORD116* snoRNA gene cluster have been found in patients with classic PWS in conjunction with normal methylation patterns in this region, thus implying that the *SNORD116* gene cluster is a minimally critical PWS region [2, 7–13]. There are some case reports that suggest that the PWS critical region is more complex than this isolated snoRNA gene cluster [14–16]. Newer evidence points to a more nuanced understanding in which both *SNORD116* and *SNURF-SNRPN* distinctly contribute to different components of the PWS phenotype [17, 18].

Here, we report a *de novo* microdeletion within 15q11.2 in a woman with classic manifestations of PWS. The deletion included only exons 2 and 3 of *SNURF-SNRPN*, and we demonstrate expression of *SNURF-SNRPN* cDNA. We also found preserved expression of several snoRNA clusters including *SNORD116*, the foremost postulated PWS critical region [13]. No expression data was conducted on other genes within the region. This case indicates that the PWS phenotype is possible through a proximal loss of *SNURF-SNRPN* and challenges the paradigm that loss of active snoRNA gene clusters, specifically *SNORD116*, is required for phenotypic manifestation of PWS.

## 2. Patient Report

**Figure 1:**
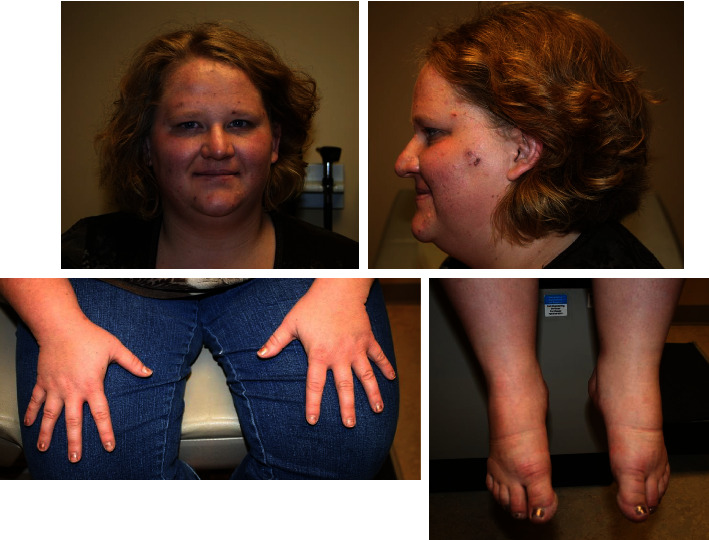
Clinical photographs of present case. Clockwise from top left: front photo of face demonstrating elevated BMI, facial features including narrow appearing face, almond-shaped eyes, thin upper lip, fair skin and hair, mild left esotropia. Feet photo demonstrating small feet. Hands photo demonstrating small hands.

A 23-year-old woman was evaluated for the first time in a medical genetics clinic for an intellectual disability. Prenatally, she was noted to have fetal polyhydramnios, followed in the neonatal period by severe hypotonia and feeding difficulties requiring syringe feeding. This continued in childhood as a persistent failure to thrive. By the age of 8 years, she developed hyperphagia, nonsatiety, and food hoarding behaviors that have continued into adulthood. She exhibited delayed puberty and mild developmental delays in all milestones with greater deficits in speech and gross motor skills. Her IQ was low-normal per clinical documentation, requiring an individualized education plan in school. During childhood, she showed signs of abnormal behaviors including sensory integration issues, skin picking, anger outbursts, and sleep difficulties (waking three times per night). She also had highly viscous saliva and experienced difficulty in articulating words, described as “mumbling.” The patient completed high school with supportive services and pursued vocational training as an adult after completing an associate degree. Family history was negative for any other individuals with signs of PWS. At clinical presentation (23 years old), her height was 150 cm (2^nd^ %ile), weight was 95.5 kg (99^th^ %ile), and body mass index (BMI) was 42.52 kg/m^2^. She was noted to have central obesity. Her skin was fair with light blonde hair. Her skin had red punctate scabs over her forearms from skin picking. Her facial features (Figure 1) included almond-shaped eyes, mild left esotropia, and a small mouth with downturned corners. She did not resemble her parents. She had small hands and feet with normal finger and toe morphology. A musculoskeletal exam revealed low tone throughout. She was otherwise neurologically typical aside from the observed skin picking, social anxiety and nervousness, and avoidance of eye contact. The patient's overall PWS clinical score was 10 (maximum of 13.5 points, Table 1), well above the clinical score criteria of 8 or greater established for diagnosis of PWS [4, 5, 19] based on the well-established definition by Holm et al. Her clinical diagnosis of classic PWS is distinguished from a diagnosis of nonclassic PWS, in which a patient has some features of PWS but does not have a clinical score of at least 8.

## 3. Materials and Methods

### 3.1. Clinical Testing

Methylation analysis for the PWS/Angelman Syndrome region was performed clinically by ARUP Laboratories (Salt Lake City, UT) using a standard methylation sensitive polymerase chain reaction/fluorescence monitoring assay (Roche Molecular Systems, Inc.). Per ARUP, this test has a sensitivity of over 99% for detecting PWS caused by methylation defects. Chromosomal microarray (CMA) was performed clinically by the Colorado Genetics Laboratory of the University of Colorado Denver on DNA extracted from patient's peripheral blood and hybridized with same-sex normal reference DNA using the CytoChip 180k Oligo Array platform (Illumina, Inc). Fluorescence in situ hybridization (FISH) analysis was performed clinically on samples from the patient and both parents by the Colorado Genetics Laboratory of the University of Colorado Denver, using BAC clone RP11-125E1, which hybridizes to the PWS region on 15q11.2. *SNURF-SNRPN* expression studies were performed at Stanford Genetics Laboratories with standard amplification of *SNURF-SNRPN* exons 9 and 10 using complementary DNA extracted from peripheral blood leukocytes [20].

The patient and her parents gave permission for the additional studies based on our genetic research protocol (#07-0516) which was reviewed and approved by the Colorado Multi-Institutional Review Board. They also provided written consent for the publication of the clinical information and photographs.

### 3.2. Molecular Analysis

Genomic DNA was isolated from white blood cells by standard methods. RNA was isolated from the patient's saliva using ORAGENE-RNA kit (cat.# RE-100) (DNA GenoTek, Kanata, Canada) according to the manufacturer protocols. RNA was DNase treated by the DNase Turbo kit (Ambion cat.#AM1907) (ThermoFisher Scientific, Waltham, MA, USA). cDNA was synthesized by the Thermo Script kit (Invitrogen cat.11146-024) (ThermoFisher Scientific) using random primers. PCR was performed using the AmpliTaq Gold kit (Applied Biosystems cat.#4311814) (ThermoFisher Scientific). Cycling conditions were 94°C for 10 min; (94°C for 30 sec; 62°C^*∗*^ for 1 min; 72°C for 1 min) for 14 cycles; (94°C for 30 sec; 55°C^*∗*^ for 1 min; 72°C for 1 min) for 36 cycles; 72°C for 7 min; 4°C on hold; primers are available upon request.

*SNURF-SNPRN* exon 2 was amplified with the following primers: SNRPNgex2F primer: 5′-CAGGGCAGGGAAAGCGAGGAGGAA-3′; SNRPNgex2R primer: 5′-TTACTGTAAAAGGAAGCAGAGCAA-3′. Single nucleotide polymorphism (SNP) genotyping was performed on DNA from the patient and both parents with Sanger sequencing of the PCR-amplified genomic DNA fragments. SNPs were detected in the patients, and her parents using the dbSNP database (Table 2).

Reverse transcription-PCR was performed using primers that spanned the coordinates chr15 : 25200167–25223718 (hg19). The start coordinate of the first primer was within intron 5 of transcript NM_001400738, and the end coordinate of the second primer was within the exon 14 of transcript NM_001400738. Primer sequences are available upon request.

*SNORD116* expression in the patient was analyzed using RNA isolated from saliva by RT-PCR using specific primers for one of the exons (HBII-85F 5′-TCGATGATGAGTCCCCCATAA; HBII-85R 5′-CCTCAGTTCCGATGAGAACGA).

**Figure 2:**
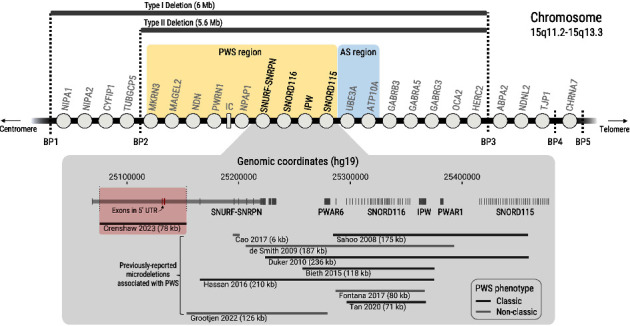
Visualization of chromosomal microdeletions at 15q11.2-q13.3 associated with PWS. The top half of this figure demonstrates a schematic of the 15q11.2-q13.3 locus (not to scale). The classic type I and II deletions (6 Mb and 5.6 Mb, respectively) associated with PWS are shown for reference as grey bars. The PWS and Angelman syndrome (AS) regions are highlighted in yellow and blue, respectively. Breakpoints (BP) are also indicated. The lower half of the figure demonstrates a 420 kb region that contains microdeletions reported in the literature that are adjacent to the deletion described in this paper. Deletions associated with a classic PWS phenotype are shown in dark grey, and those associated with a nonclassic PWS phenotype are shown in light grey. The red box denotes the location of the deletion described in this paper, which includes exons 2 and 3 of *SNRPN* (NM_001400738.1). The gene symbols associated with select transcripts of clinical importance are displayed. The term “*SNURF-SNRPN*” is utilized here for consistency with prior literature and reflects the bicistronic nature of the transcript.

In Figure 2, hg19 RefSeq-curated transcripts with exon coordinates on chromosome 15 were downloaded from the UCSC genome browser and loaded into R version 4.0.3. Visualizations of the data were generated using ggplot2 version 3.3.5. Exons overlapping with the coordinates spanning any of the deletions listed in the lower half of the figure were then selected. RefSeq accession numbers were subsequently mapped to gene symbols using the gene table from NCBI datasets (https://www.ncbi.nlm.nih.gov/datasets/tables/genes/). To ensure that all gene locations were mapped in a standardized manner, the longest RefSeq transcript was selected and then visualized. Select transcripts of clinical importance were annotated in Figure 2. A list of the transcripts utilized in this visualization is available in Supplementary Table 1.

## 4. Results

**Figure 3:**
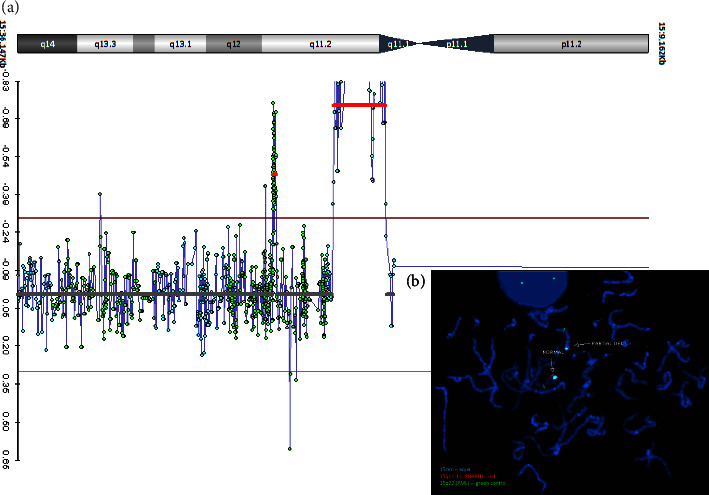
Fluorescence in situ hybridization (FISH) and chromosomal microarray. (a). Chromosomal microarray results of proband indicating ∼132 kb deletion at 15q11.2 (red line). (b). Analysis by FISH with cloneRP11-125E1 (red signal) confirmed partial deletion at 15q11.2. Blue probe is the centromere of chromosome 15, and the green probe is the control PML probe of 15q22. FISH, fluorescence in situ hybridization.

**Figure 4:**
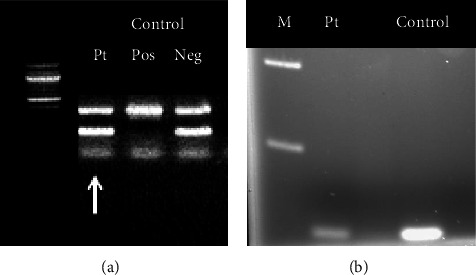
SNURF-SNRPN exons 9 and 10 and SNORD116. (a). Analysis using established clinical expression reverse transcribed RNA assay in peripheral lymphoblasts showing normal expression of *SNRPN* exons 9 and 10 in the patient (column labeled Pt and with arrow). Top band is control gene product (WASP), second band is *SNRPN* exons 9 and 10 as described in previous literature [20]. Columns from left to right: ladder, present case, positive control (patient with PWS without *SNRPN* exons 9 and 10), and negative control (patient who does not have PWS with present *SNRPN* exons 9 and 10). (b). Demonstration of *SNORD116* expression in patient. Columns from left to right: markers, present case, and control (patient who does not have PWS). Lowest band is *SNORD116*. Pt, patient; Pos, positive; Neg, negative; M, markers; PWS, Prader-Willi syndrome.

Molecular studies in this patient diagnosed with classic PWS revealed a normal PWS methylation pattern and an atypical 15q11.2 deletion by CMA analysis. Based on the CMA (Figure 3(a)), the deletion size was initially estimated at 132 kb and spanned linear positions 25,092,034−25,224,089 (NCBI human genome reference assembly Build 37 (hg19)). The deletion was confirmed by FISH (Figure 3(b)) to be present in the patient (by a diminished signal consistent with a partially adherent FISH probe) and absent in both parents (fully adherent FISH probe). Sequencing and SNP mapping refined the deletion and narrowed the region to 78 kb between two heterozygous *SNURF-SNRPN* SNPs: a novel SNP in intron 2 and the rs61999138 SNP. The novel SNP was detected in both parents. Gene expression analysis of *SNURF-SNRPN* in cDNA from the patient's peripheral blood lymphoblasts with a clinical assay revealed intact exons 9 and 10 (Figure 4(a)). In addition, RT-PCR molecular studies from the patient's saliva demonstrated preserved expression of *SNURF-SNRPN* exons 6 through 13, as well as *PAR5* and *SNORD116*. *SNORD116* expression in the patient was confirmed with targeted RT-PCR and gel electrophoresis (Figure 4(b)). Figure 2 demonstrates the novel location of the deletion detected in this patient with classic PWS, the regions implicated, and other regions of interest as outlined in the literature.

## 5. Discussion

Our case provides additional evidence of the complexity of the minimal critical deletion regions that result in PWS. Our patient presented with most of the major and minor characteristics observed clinically in PWS (Table 1). A previously undescribed microdeletion was identified in the 15q11.2 region [4, 5]. Molecular tests revealed a normal methylation pattern at the SNRPN locus, but CMA and SNP homozygosity analysis revealed a 78 kb deletion that included *SNURF-SNRPN* exons 2 and 3. FISH analysis targeting the 78 kb region confirmed a deletion in the patient; parental studies showed a normal FISH pattern and no deletion. This small deletion size (78 kb) contrasts with the larger type I and type II deletions classically associated with PWS that are 6.0 Mb and 5.6 Mb, respectively [10]. Type I and II deletions can usually be detected by testing for the absence of expression of SNURF-SNRPN exons 9 and 10. In the patient described here, exons 9 and 10 of these genes in addition to the *SNORD116* gene cluster were present and expressed. Importantly, a diagnosis of PWS may be missed if testing stopped with a negative test targeted for SNURF-SNURPN exons 9 and 10. If clinical presentation is consistent for PWS and a routine clinical test is negative, it is critical to pursue additional testing, such as CMA, to evaluate for less common genetic causes of PWS.

Ultimately, it has been challenging to determine the exact genes implicated in the pathogenesis of PWS. However, one leading theory is that *SNORD116* is the minimal critical region for the PWS phenotype [13]. This was ascertained by determining the smallest overlapping region of atypical deletions found in patients with classic PWS [2, 8–11]. Deletions described in the literature that include *SNORD116* and are associated with PWS only sometimes include *SNURF-SNRPN.* Of the eight deletions reported that include *SNORD116,* three also include all or part of *SNURF-SNRPN* [7, 9, 10]. These three studies that included *SNURF-SNRPN* reported patients with a wide array of symptoms, from those with only a few features of PWS to those with classic PWS. In addition, the five studies that report deletions including *SNORD116* but sparing *SNURF-SNRPN* also report a wide spectrum of phenotypes associated with these deletions [2, 8, 11, 13, 21]. Thus, there does not seem to be a genotype-phenotype correlation among patients with deletions that spare *SNURF-SNRPN.*

Despite this replicated evidence, other reports point to a more complex picture, especially when exons near the imprinting center are affected. For example, one of the smallest atypical deletions is described in a patient with many of the PWS characteristics [14]. They describe a 6.4 kb deletion that overlaps with the imprinting center in a patient with decreased fetal movement, poor feeding, and hypotonia in infancy who developed developmental delays and obesity with small hands and feet (consistent with nonclassic PWS given that the clinical score was less than 8). In another case series (*N* = 8) of atypical deletions in patients with PWS, one of the patients also had a partial deletion of *SNURF-SNRPN* (including the entire *SNURF* transcript (NM_005678.5) and exons 4–13 of SNRPN (NM_001400738.1); see Figure 2) that did not directly affect *SNORD116* [18]. This patient had infantile hypotonia, obesity, hyperphagia, behavioral challenges (irritability), and skin-picking and did not require tube feeding as an infant; thus, this patient also did not meet enough criteria to be consistent with classic PWS.

There are two reports of patients with variants in *SNURF-SNRPN* who have features of PWS. The first is a patient with a missense variant in *SNURF-SNRPN* (c.193C > T, p.Arg65Trp) as well as a high degree of homozygosity that affected many genes in addition to part of *SNURF-SNRPN* [15]. This patient had hypotonia and poor feeding in infancy with decreased fetal movement and developed hyperphagia, obesity, small hands, and endocrine abnormalities. Functional studies show the overexpression of *SNURF-SNRPN,* possibly resulting in a dominant negative effect, but they also demonstrate normal RNA expression of *SNORD116* [15]. Huang et al. also recently reported a mosaic, nonsense *SNRPN* variant (c.73C > T, p.R25X) found in a patient with some findings of classic PWS, although with an overall milder form of the phenotype [17]. The present study is now the fourth published patient with classic PWS findings that does not demonstrate an aberration of either an exon near the imprinting center of *SNURF-SNRPN* nor *SNORD116* [15, 17, 18]. The deletion found in our patient is also unique without any overlapping deletion reported in the literature.

## 6. Conclusion

We demonstrate a unique molecular presentation in a patient with classic PWS associated with a 78 kb microdeletion involving exons 2-3 of the *SNURF-SNRPN* gene and preserved expression of downstream *SNORD116*. This result shows that classic PWS is not solely dependent on absent *SNORD116* expression and that more work is needed to understand the mechanisms driving the phenotype. This study further demonstrates the importance of considering atypical microdeletions as a mechanism of PWS when initial methylation studies are normal. Our work also highlights that both methylation testing and CMA may be needed to ensure that a molecular diagnosis of PWS is not missed, particularly when clinical suspicion is high.

## Figures and Tables

**Table 1:** Clinical features present in the patient (10 points).

	Criteria from Holm et al., 1993	Criteria to prompt DNA testing from Gunay-Aygun et al., 2001	Present case
Major criteria (1 point)	Neonatal/infantile central hypotonia, poor suck	X	X
	Feeding problems in infancy requiring special techniques		X
	Excessive/rapid weight gain between 1-6yo		
	Characteristic facial features		X
	Hypogonadism	X	
	Global developmental delay	X	X
	Hyperphagia/food foraging, central obesity	X	X
	Deletion 15q11-13		

Minor criteria (1/2 point)	Decreased fetal movement/infantile lethargy/weak cry in infancy		X
	Characteristic behavior challenges	X	X
	Sleep disturbance/sleep apnea		X
	Short stature		X
	Hypopigmentation		X
	Small hands/feet		X
	Narrow hands		
	Eye abnormalities		X
	Thick viscous saliva		X
	Speech articulation defects		X
	Skin picking		X

Supportive findings	High pain threshold		
	Decreased vomiting		X
	Temperature instability/sensitivity		
	Scoliosis/kyphosis		
	Early adrenarche		
	Osteoporosis		
	Unusual skill with jigsaw puzzles		
	Normal neuromuscular studies		

Modified from Holm et al. 1993 (8 points needed to meet diagnostic criteria, patient score = 10) [4, 5].

**Table 2:** Single nucleotide polymorphism (SNP) analysis data.

SNPs	Patient	Father	Mother
Chr15 : 24,830,384 (build hg38)	GA	GA	GA
rs61994705	GG	GC	GC
rs187852468	GG	GC	GC
rs11161153	GG	GG	GC
rs71461569	CC	AC	AC
rs61999138	TC	CC	TC

SNPs interrogated in both the patient and her parents to further narrow the smallest possible deleted region in the patient presented.

## Data Availability

Additional data beyond what are in the manuscript that support the results of the study can be requested from the authors at any time.

## Supplementary Materials

Supplementary Materials Supplementary 1: A list of all of the 102 Refseq exons utilized in the visualization shown in Figure 2 is available in Supplementary table 1. Each row in the table corresponds to a single exon. The descriptions of the columns in the table are as follows. “Ref_genome” refers to the reference genome used (in this case, hg19). “Chrom” refers to the chromosome (in this case, all genes were located on chromosome 15). “Start” refers to the first base of the exon, and “Stop” refers to the last base of the exon. “Exon_len” refers to the length of each exon. “Refseq” refers to the refseq accession number that contains the indicated exon. “Is_within_cren_del” is either true or false and refers to whether the indicated exon is found in the deletion described in the patient in this paper. In this case, there were two exons of the SNRPN gene (NM_001400738.1) that were found within this deletion. “Exon_number” corresponds to the exon number for the corresponding transcript (eg “1” corresponds to the 1st exon, “2” corresponds to the 2nd exon, etc.). Note that some genes may only have 1 exon. ()
