# Supplementary material for: An Atypical 15q11.2 Microdeletion Not Involving SNORD116 Resulting in Prader–Willi Syndrome
Source: Case Rep Genet. 2023 Sep 13;2023:4225092. doi: 10.1155/2023/4225092 (PMC10511293; doi:10.1155/2023/4225092)
Supplement: Supplementary Materials — Supplementary 1: A list of all of the 102 Refseq exons utilized in the visualization shown in Figure 2 is available in Supplementary table 1. Each row in the table corresponds to a single exon. The descriptions of the columns in the table are as follows. “Ref_genome” refers to the reference genome used (in this case, hg19). “Chrom” refers to the chromosome (in this case, all genes were located on chromosome 15). “Start” refers to the first base of the exon, and “Stop” refers to the last base of the exon. “Exon_len” refers to the length of each exon. “Refseq” refers to the refseq accession number that contains the indicated exon. “Is_within_cren_del” is either true or false and refers to whether the indicated exon is found in the deletion described in the patient in this paper. In this case, there were two exons of the SNRPN gene (NM_001400738.1) that were found within this deletion. “Exon_number” corresponds to the exon number for the corresponding transcript (eg “1” corresponds to the 1st exon, “2” corresponds to the 2nd exon, etc.). Note that some genes may only have 1 exon. () [file 4225092.f1.docx]

| Supplementary 1 Table | | | | | | | | |
| --- | --- | --- | --- | --- | --- | --- | --- | --- |
| **ref_genome** | **chrom** | **start** | **stop** | **exon_len** | **refseq** | **symbol** | **is_within_cren_del** | **exon_number** |
| **hg19** | chr15 | 25068784 | 25068970 | 187 | NM_001400738.1 | SNRPN | FALSE | 1 |
| **hg19** | chr15 | 25131663 | 25131736 | 74 | NM_001400738.1 | SNRPN | TRUE | 2 |
| **hg19** | chr15 | 25133277 | 25133987 | 711 | NM_001400738.1 | SNRPN | TRUE | 3 |
| **hg19** | chr15 | 25165158 | 25165271 | 114 | NM_001400738.1 | SNRPN | FALSE | 4 |
| **hg19** | chr15 | 25195722 | 25195837 | 116 | NM_001400738.1 | SNRPN | FALSE | 5 |
| **hg19** | chr15 | 25200134 | 25200209 | 76 | NM_005678.5 | SNURF | FALSE | 1 |
| **hg19** | chr15 | 25207261 | 25207356 | 96 | NM_001400738.1 | SNRPN | FALSE | 6 |
| **hg19** | chr15 | 25207261 | 25207356 | 96 | NM_005678.5 | SNURF | FALSE | 2 |
| **hg19** | chr15 | 25213079 | 25213229 | 151 | NM_001400738.1 | SNRPN | FALSE | 7 |
| **hg19** | chr15 | 25213079 | 25213229 | 151 | NM_005678.5 | SNURF | FALSE | 3 |
| **hg19** | chr15 | 25219458 | 25219603 | 146 | NM_001400738.1 | SNRPN | FALSE | 8 |
| **hg19** | chr15 | 25219458 | 25219603 | 146 | NM_005678.5 | SNURF | FALSE | 4 |
| **hg19** | chr15 | 25220505 | 25220656 | 152 | NM_001400738.1 | SNRPN | FALSE | 9 |
| **hg19** | chr15 | 25220505 | 25220656 | 152 | NM_005678.5 | SNURF | FALSE | 5 |
| **hg19** | chr15 | 25221452 | 25221563 | 112 | NM_001400738.1 | SNRPN | FALSE | 10 |
| **hg19** | chr15 | 25221452 | 25221563 | 112 | NM_005678.5 | SNURF | FALSE | 6 |
| **hg19** | chr15 | 25222024 | 25222176 | 153 | NM_001400738.1 | SNRPN | FALSE | 11 |
| **hg19** | chr15 | 25222024 | 25222176 | 153 | NM_005678.5 | SNURF | FALSE | 7 |
| **hg19** | chr15 | 25222925 | 25223063 | 139 | NM_001400738.1 | SNRPN | FALSE | 12 |
| **hg19** | chr15 | 25222925 | 25223063 | 139 | NM_005678.5 | SNURF | FALSE | 8 |
| **hg19** | chr15 | 25223340 | 25223465 | 126 | NM_001400738.1 | SNRPN | FALSE | 13 |
| **hg19** | chr15 | 25223340 | 25223465 | 126 | NM_005678.5 | SNURF | FALSE | 9 |
| **hg19** | chr15 | 25223554 | 25223870 | 317 | NM_001400738.1 | SNRPN | FALSE | 14 |
| **hg19** | chr15 | 25223554 | 25223870 | 317 | NM_005678.5 | SNURF | FALSE | 10 |
| **hg19** | chr15 | 25227141 | 25227215 | 75 | NR_001293.1 | SNORD107 | FALSE | 1 |
| **hg19** | chr15 | 25227141 | 25228937 | 1797 | NR_022011.1 | PWARSN | FALSE | 1 |
| **hg19** | chr15 | 25230007 | 25233379 | 3373 | NR_022008.1 | PWAR5 | FALSE | 1 |
| **hg19** | chr15 | 25230247 | 25230313 | 67 | NR_001294.1 | SNORD64 | FALSE | 1 |
| **hg19** | chr15 | 25232072 | 25232140 | 69 | NR_001292.2 | SNORD108 | FALSE | 1 |
| **hg19** | chr15 | 25277020 | 25281637 | 4618 | NR_146168.1 | PWAR6 | FALSE | 1 |
| **hg19** | chr15 | 25287121 | 25287187 | 67 | NR_001295.1 | SNORD109A | FALSE | 1 |
| **hg19** | chr15 | 25296623 | 25296719 | 97 | NR_003316.1 | SNORD116-1 | FALSE | 1 |
| **hg19** | chr15 | 25299356 | 25299452 | 97 | NR_003317.1 | SNORD116-2 | FALSE | 1 |
| **hg19** | chr15 | 25302006 | 25302102 | 97 | NR_003318.1 | SNORD116-3 | FALSE | 1 |
| **hg19** | chr15 | 25304684 | 25304781 | 98 | NR_003319.1 | SNORD116-4 | FALSE | 1 |
| **hg19** | chr15 | 25307479 | 25307575 | 97 | NR_003320.1 | SNORD116-5 | FALSE | 1 |
| **hg19** | chr15 | 25310172 | 25310269 | 98 | NR_003321.1 | SNORD116-6 | FALSE | 1 |
| **hg19** | chr15 | 25312934 | 25313030 | 97 | NR_003322.1 | SNORD116-7 | FALSE | 1 |
| **hg19** | chr15 | 25315578 | 25315674 | 97 | NR_003323.1 | SNORD116-8 | FALSE | 1 |
| **hg19** | chr15 | 25318253 | 25318349 | 97 | NR_003324.1 | SNORD116-9 | FALSE | 1 |
| **hg19** | chr15 | 25319260 | 25319363 | 104 | NR_003325.1 | SNORD116-10 | FALSE | 1 |
| **hg19** | chr15 | 25321075 | 25321168 | 94 | NR_003326.2 | SNORD116-11 | FALSE | 1 |
| **hg19** | chr15 | 25322197 | 25322290 | 94 | NR_003327.2 | SNORD116-12 | FALSE | 1 |
| **hg19** | chr15 | 25324204 | 25324297 | 94 | NR_003328.2 | SNORD116-13 | FALSE | 1 |
| **hg19** | chr15 | 25325288 | 25325381 | 94 | NR_003329.2 | SNORD116-14 | FALSE | 1 |
| **hg19** | chr15 | 25326433 | 25326526 | 94 | NR_003330.2 | SNORD116-15 | FALSE | 1 |
| **hg19** | chr15 | 25327914 | 25328007 | 94 | NR_003331.2 | SNORD116-16 | FALSE | 1 |
| **hg19** | chr15 | 25328734 | 25328827 | 94 | NR_003332.1 | SNORD116-17 | FALSE | 1 |
| **hg19** | chr15 | 25330531 | 25330624 | 94 | NR_003333.2 | SNORD116-18 | FALSE | 1 |
| **hg19** | chr15 | 25331673 | 25331766 | 94 | NR_001290.2 | SNORD116-19 | FALSE | 1 |
| **hg19** | chr15 | 25332808 | 25332901 | 94 | NR_003334.2 | SNORD116-20 | FALSE | 1 |
| **hg19** | chr15 | 25332808 | 25332901 | 94 | NR_003334.2 | SNORD116-20 | FALSE | 1 |
| **hg19** | chr15 | 25333950 | 25334043 | 94 | NR_003335.2 | SNORD116-21 | FALSE | 1 |
| **hg19** | chr15 | 25333950 | 25334043 | 94 | NR_003335.2 | SNORD116-21 | FALSE | 1 |
| **hg19** | chr15 | 25335069 | 25335162 | 94 | NR_003336.2 | SNORD116-22 | FALSE | 1 |
| **hg19** | chr15 | 25336932 | 25337025 | 94 | NR_003337.2 | SNORD116-23 | FALSE | 1 |
| **hg19** | chr15 | 25339183 | 25339276 | 94 | NR_003338.2 | SNORD116-24 | FALSE | 1 |
| **hg19** | chr15 | 25342809 | 25342902 | 94 | NR_003339.2 | SNORD116-25 | FALSE | 1 |
| **hg19** | chr15 | 25344645 | 25344742 | 98 | NR_003340.2 | SNORD116-26 | FALSE | 1 |
| **hg19** | chr15 | 25346721 | 25346814 | 94 | NR_003341.2 | SNORD116-27 | FALSE | 1 |
| **hg19** | chr15 | 25349788 | 25349880 | 93 | NR_003361.1 | SNORD116-28 | FALSE | 1 |
| **hg19** | chr15 | 25351667 | 25351751 | 85 | NR_003360.1 | SNORD116-29 | FALSE | 1 |
| **hg19** | chr15 | 25353415 | 25353499 | 85 | NR_047032.1 | SNORD116-30 | FALSE | 1 |
| **hg19** | chr15 | 25361692 | 25362662 | 971 | NR_023915.1 | IPW | FALSE | 1 |
| **hg19** | chr15 | 25363415 | 25363529 | 115 | NR_023915.1 | IPW | FALSE | 2 |
| **hg19** | chr15 | 25364212 | 25367623 | 3412 | NR_023915.1 | IPW | FALSE | 3 |
| **hg19** | chr15 | 25380789 | 25383200 | 2412 | NR_022009.1 | PWAR1 | FALSE | 1 |
| **hg19** | chr15 | 25415870 | 25415951 | 82 | NR_001291.2 | SNORD115-1 | FALSE | 1 |
| **hg19** | chr15 | 25417782 | 25417863 | 82 | NR_003294.1 | SNORD115-2 | FALSE | 1 |
| **hg19** | chr15 | 25420074 | 25420155 | 82 | NR_003295.1 | SNORD115-3 | FALSE | 1 |
| **hg19** | chr15 | 25421979 | 25422060 | 82 | NR_003296.1 | SNORD115-4 | FALSE | 1 |
| **hg19** | chr15 | 25423885 | 25423966 | 82 | NR_003297.1 | SNORD115-5 | FALSE | 1 |
| **hg19** | chr15 | 25425644 | 25425725 | 82 | NR_003298.1 | SNORD115-6 | FALSE | 1 |
| **hg19** | chr15 | 25427532 | 25427613 | 82 | NR_003299.1 | SNORD115-7 | FALSE | 1 |
| **hg19** | chr15 | 25429453 | 25429534 | 82 | NR_003300.1 | SNORD115-8 | FALSE | 1 |
| **hg19** | chr15 | 25430778 | 25430859 | 82 | NR_003301.1 | SNORD115-9 | FALSE | 1 |
| **hg19** | chr15 | 25432683 | 25432763 | 81 | NR_003302.1 | SNORD115-10 | FALSE | 1 |
| **hg19** | chr15 | 25434561 | 25434642 | 82 | NR_003303.1 | SNORD115-11 | FALSE | 1 |
| **hg19** | chr15 | 25436563 | 25436644 | 82 | NR_003304.1 | SNORD115-12 | FALSE | 1 |
| **hg19** | chr15 | 25438468 | 25438549 | 82 | NR_003305.1 | SNORD115-13 | FALSE | 1 |
| **hg19** | chr15 | 25440068 | 25440148 | 81 | NR_003306.1 | SNORD115-14 | FALSE | 1 |
| **hg19** | chr15 | 25442723 | 25442803 | 81 | NR_003307.1 | SNORD115-15 | FALSE | 1 |
| **hg19** | chr15 | 25444595 | 25444676 | 82 | NR_003308.1 | SNORD115-16 | FALSE | 1 |
| **hg19** | chr15 | 25446470 | 25446551 | 82 | NR_003309.1 | SNORD115-17 | FALSE | 1 |
| **hg19** | chr15 | 25448374 | 25448455 | 82 | NR_003310.1 | SNORD115-18 | FALSE | 1 |
| **hg19** | chr15 | 25449504 | 25449585 | 82 | NR_003311.1 | SNORD115-19 | FALSE | 1 |
| **hg19** | chr15 | 25451409 | 25451490 | 82 | NR_003312.1 | SNORD115-20 | FALSE | 1 |
| **hg19** | chr15 | 25453230 | 25453310 | 81 | NR_003313.1 | SNORD115-21 | FALSE | 1 |
| **hg19** | chr15 | 25455065 | 25455146 | 82 | NR_003314.1 | SNORD115-22 | FALSE | 1 |
| **hg19** | chr15 | 25456839 | 25457180 | 342 | NR_022010.1 | PWAR4 | FALSE | 1 |
| **hg19** | chr15 | 25456943 | 25457024 | 82 | NR_003315.1 | SNORD115-23 | FALSE | 1 |
| **hg19** | chr15 | 25458806 | 25458876 | 71 | NR_003495.1 | SNORD115-24 | FALSE | 1 |
| **hg19** | chr15 | 25460688 | 25460769 | 82 | NR_003342.1 | SNORD115-25 | FALSE | 1 |
| **hg19** | chr15 | 25463764 | 25463845 | 82 | NR_003343.1 | SNORD115-26 | FALSE | 1 |
| **hg19** | chr15 | 25465650 | 25465725 | 76 | NR_003496.1 | SNORD115-27 | FALSE | 1 |
| **hg19** | chr15 | 25467501 | 25467574 | 74 | NR_003497.1 | SNORD115-28 | FALSE | 1 |
| **hg19** | chr15 | 25468393 | 25468474 | 82 | NR_003344.1 | SNORD115-29 | FALSE | 1 |
| **hg19** | chr15 | 25470350 | 25470431 | 82 | NR_003345.1 | SNORD115-30 | FALSE | 1 |
| **hg19** | chr15 | 25472256 | 25472337 | 82 | NR_003346.1 | SNORD115-31 | FALSE | 1 |
| **hg19** | chr15 | 25474114 | 25474195 | 82 | NR_003347.1 | SNORD115-32 | FALSE | 1 |
| **hg19** | chr15 | 25475985 | 25476066 | 82 | NR_003348.1 | SNORD115-33 | FALSE | 1 |
| **hg19** | chr15 | 25477534 | 25477615 | 82 | NR_003349.1 | SNORD115-34 | FALSE | 1 |
